# Evaluation of the Antimicrobial, Cyto-Genotoxic, and Antigenotoxic Activity of *Dipteryx odorata* Seed Extracts

**DOI:** 10.3390/ijms27020561

**Published:** 2026-01-06

**Authors:** Margarita Dormousoglou, Ioannis Galiatsatos, Panagiota Stathopoulou, Regina Fragkouli, Maria Antonopoulou, Damian E. L. Fetzer, Marcos L. Corazza, Vasilios Triantafylidis, George Tsiamis, Dimitris Vlastos, Ioanna Efthimiou

**Affiliations:** 1 Department of Sustainable Agriculture, University of Patras, 30131 Agrinio, Greecepanstath@upatras.gr (P.S.); gtsiamis@upatras.gr (G.T.); 2 Department of Food Science & Technology, University of Patras, 30100 Agrinio, Greece; 3 Department of Biological and Chemical Engineering, Aarhus University, 8200 Aarhus, Denmark; 4 Department of Chemical Engineering, Federal University of Paraná, Curitiba 81531-990, Brazil; 5 Department of Biology, University of Patras, 26500 Patras, Greece

**Keywords:** tonka beans, antimicrobial activity, cytotoxicity, genotoxicity, antigenotoxicity, CBMN

## Abstract

In the present study, *Dipteryx odorata* seeds (tonka beans) were extracted via the Soxhlet method to acquire ethanolic (TBSE) and hexane (TBSH) extracts. Both extracts were characterized using Gas Chromatography–Mass Spectrometry (GC-MS). The antimicrobial activity was evaluated against two Gram-positive (*Bacillus licheniformis*, *Staphylococcus epidermidis*) and two Gram-negative (*Escherichia coli*, *Pseudomonas aeruginosa*) human pathogens using the disc diffusion test (DDT), followed by the determination of Minimum Inhibitory Concentrations (MIC). The Cytokinesis-Block Micronucleus (CBMN) assay was applied in human lymphocytes (0.1, 0.2, 0.5 µL/mL), to investigate the cyto-genotoxic activity of both extracts, while their antigenotoxic potential was evaluated against mitomycin C (MMC) (0.5 μg/mL). Coumarin was the major constituent in both extracts. TBSE exhibited remarkable antimicrobial activity, whereas TBSH was not equally potent. Cytotoxicity was reported for higher doses, while no genotoxicity was observed, except for 0.2 µL/mL for TBSE. A considerable antigenotoxic activity was shown by the lowest dose of TBSE, which was not present at the two highest concentrations. However, TBSH did not diminish the MMC mediated genotoxicity, while at the same time led to an increase in genotoxic potency. To our knowledge, this is the first comprehensive evaluation of the cyto-genotoxic and antigenotoxic profiles of tonka bean extracts.

## 1. Introduction

Natural products and their extracts have been widely used since ancient times in everyday life applications and as remedies for several ailments. Their significant potential and several beneficial properties, in combination with their low toxicity have highlighted them as valuable candidates in drug manufacturing and as promising therapeutics [1]. The Amazon is a biodiversity hotspot, possessing a plethora of plants that have been used in traditional medicine by indigenous peoples, with many of them being evaluated for pharmaceutical applications [2]. Plants contain numerous active phytochemicals commonly called secondary metabolites which are significant sources for drug discovery [3,4]. The chemical diversity of phytochemicals has attracted researchers to develop effective medicines against human disorders [5].

*Dipteryx odorata* (commonly known as tonka bean tree or *cumaru*) is a tall arboreal species, belonging to the Fabaceae family and is native to the central and northern parts of South America including the Amazonian rainforests [6]. Venezuela and Brazil are the main commercial producers [7]. *D. odorata* holds ethnobotanical importance in traditional Amazonian medicine, where it has been employed against respiratory ailments, inflammation, wounds, fever, and digestive disorders among others [8].

It has high economic value because of its seeds (tonka beans), which are rich in coumarin (1,2-benzopyrone), a chemical compound that imparts a distinctive aroma of vanilla, almond, and cinnamon [9]. As a result, this plant has been widely used in gastronomy, perfumery and aromatherapy [8,10]. Tonka beans and their extracts possess several therapeutic properties including antimicrobial, antioxidant, antifungal, antiviral, antiproliferative and anti-inflammatory [11], with coumarin usually ranging from 1–3% weight/seed. Besides coumarin, the seeds contain flavonoids, phenolic acids, and related derivatives with significant antimicrobial and antioxidant activities [12,13].

Despite these promising therapeutic applications, the safety profile of tonka beans warrants careful consideration. The high percentage of coumarin is a source of concern due to its toxicity, including hepatotoxicity, which has led to regulatory restrictions on food applications [14]. Regulatory bodies, such as the European Food Safety Authority (EFSA), have established maximum dose of coumarin in edible products to avoid potential health risks [15,16]. Although *D. odorata* has been extensively studied for its timber value and forestry management, comprehensive investigations of its phytochemical composition, toxicity profile, and beneficial properties remain limited. More critically, no study to date has evaluated the cyto-genotoxic and antigenotoxic potential of tonka bean extracts, representing a significant knowledge gap given their widespread commercial use.

In the present study, a multidisciplinary approach was undertaken. Specifically, ethanol and hexane extracts (TBSE and TBSH, respectively) were derived from tonka beans via Soxhlet extraction, a simple extraction method widely used for extracting bioactive compounds from natural products due to achieving high yields, while the bioactivity of the extracted molecules remains unaffected [17,18]. Subsequently, Gas Chromatography–Mass Spectrometry (GC-MS), was used to identify the chemical constituents of the extracts. Four human pathogens, i.e., *Escherichia coli*, *Pseudomonas aeruginosa*, *Bacillus licheniformis*, and *Staphylococcus epidermidis*, were used to investigate the antimicrobial properties of the extracts using the disc diffusion test (DDT), while the microdilution method in 96-well microplates was applied to determine the Minimum Inhibitory Concentrations (MIC). Considering the cytotoxic and anticancer effects of coumarin [11]—the major compound of tonka beans—their cyto-genotoxic profile was assessed in order to perform a safety evaluation of their extracts, in addition to their antigenotoxic potential against mitomycin C (MMC) (0.5 μg/mL), a widely used mutagenic agent [19], via the application of the Cytokinesis-Block Micronucleus (CBMN) assay—a robust, simple, reliable and well-established assay—in cultured human lymphocytes at concentrations of 0.1, 0.2, and 0.5 µL/mL.

## 2. Results

### 2.1. Identification of Chemical Constituents in Tonka Bean Extracts by Gas Chromatography-Mass Spectrometry

Τhe chemical composition of the of the obtained extracts was investigated using Gas Chromatography-Mass Spectrometry (GC-MS). Τhe constituents were identified by comparing their mass spectra with the NIST library database. The results, summarized in Table 1, revealed the presence of 9 compounds in both TBSE and TBSH extracts. As expected, coumarin (C_9_H_6_O_2_) was the predominant constituent, accounting for 90.31% and 83.97% of the total peak area in TBSE and TBSH, respectively. In addition to coumarin, the extracts were characterized by the presence of fatty acids and their derivatives, as well as minor amounts of aliphatic hydrocarbons.

### 2.2. Evaluation of Antimicrobial Activity of Tonka Bean Extracts

#### 2.2.1. Antimicrobial Assessment by the Disc Diffusion Method

The disc diffusion method (Kirby-Bauer) was used for the initial, qualitative evaluation of the antimicrobial activity of the two extracts against four bacterial strains.

The results showed that TBSE exhibited significant inhibitory activity against the Gram-negative bacteria *E. coli* and *P. aeruginosa*, with an average inhibition zone diameter of 18 mm and 11.5 mm, respectively (Table 2). No inhibition zones were observed for *B. licheniformis* and *S. epidermidis*.

In contrast, TBSH did not exhibit any visible antimicrobial activity (0 mm zone of inhibition) against any of the four bacterial strains (Table 2).

#### 2.2.2. Determination of Minimum Inhibitory Concentration (MIC)

For the quantitative evaluation of antimicrobial activity, the Minimum Inhibitory Concentration (MIC) was determined using the microdilution method in 96-well plates. Bacterial viability was assessed using a resazurin indicator, where the retention of purple color indicated growth inhibition, whereas the change to pink indicates bacterial growth. The results showed that both extracts exhibited antimicrobial activity, with TBSE being more potent. Against *E. coli* TBSE inhibited growth up to a concentration of 15.6 mg/mL while TBSH inhibited growth up to a concentration of 125 mg/mL. Against *P. aeruginosa* TBSE showed the strongest activity, inhibiting growth up to a concentration of 15.6 mg/mL while TBSH inhibited growth only at the highest concentration hence the MIC value was determined to be 500 mg/mL. Against *S. epidermidis*, TBSE inhibited growth up to a concentration of 62.5 mg/mL while TBSH showed no inhibitory activity. The MIC was determined to be >500 mg/mL. Against *B. licheniformis* TBSE inhibited growth up to a concentration of 15.6 mg/mL, whereas TBSH showed no inhibitory activity. The percentages refer to the final concentrations of the extract in each well of the plate, as generated by the standard serial dilution method. The table below summarizes the data for all bacterial strains for both methods and both extracts (Table 3).

#### 2.2.3. Antimicrobial Profile of Tonka Bean Extracts

**Figure 1:**
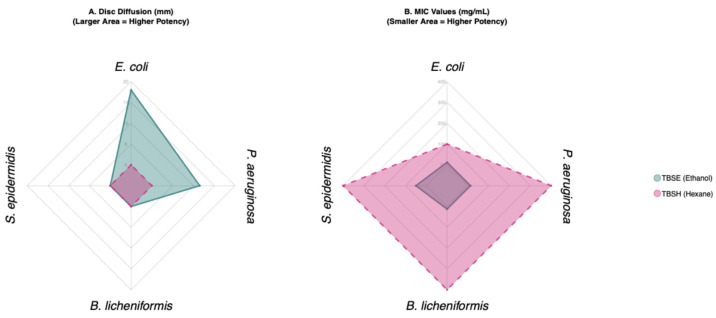
Antimicrobial efficacy of ethanol and hexane extracts. Radar charts illustrate the comparative activity of TBSE and TBSH against four bacterial strains (*E. coli*, *P. aeruginosa*, *S. epidermidis*, and *B. licheniformis*). (**A**) Mean inhibition zone diameter (mm) obtained from disc diffusion assays; larger shaded areas indicate higher antimicrobial potency. (**B**) Minimum Inhibitory Concentration (MIC) values expressed in mass concentration (mg/mL); smaller shaded areas (closer to the center) indicate higher potency (lower effective dose). TBSE (light blue) demonstrates broad-spectrum activity with high inhibition zones and low MIC values, whereas TBSH (pink) exhibits negligible activity.

The following graph shows the spectrum of antimicrobial activity of the two extracts tested. Spiderweb diagrams show the “profile” of the activity of each extract. A larger, and more extensive area indicates a stronger and broader spectrum of activity. The ethanol extract (light blue) was active, while the hexane extract (pink) is almost inactive in the determination of the zone of inhibition (Figure 1A). The shape of the polygon indicates the specificity. For example, in the MIC diagram, TBSE is equally potent against *E. coli*, *P. aeruginosa* and *B. licheniformis* (Figure 1B).

### 2.3. CBMN Assay in Human Lymphocytes

#### 2.3.1. Cyto-Genotoxic and Antigenotoxic Profile of Tonka Bean Extracts

The cytotoxic and genotoxic potential of TBSE and TBSH were evaluated at concentrations of 0.1, 0.2, and 0.5 μL/mL.

Regarding cytotoxicity, the extract exhibited a mild reduction in cell proliferation (Cytokinesis Block Proliferation Index, CBPI) at the two highest concentrations (0.2 and 0.5 μL/mL) compared to the control, with % cytostasis values of 17.5 and 15.6, respectively. As expected, MMC induced high cytotoxicity with a CBPI of 1.52. In the presence of MMC, statistically significant reduction compared to the negative control was observed in all cases, which was mild in the case of the two lowest concentrations, i.e., 0.1 and 0.2 μL/mL whereas the highest had a CBPI of 1.4, with approximately 50% cytostasis. In terms of genotoxicity, TBSE induced a statistically significant increase in micronuclei (MN) frequency only at 0.2 μL/mL, with slightly higher MN values than the negative control. No genotoxic activity was observed for the remaining concentrations. Regarding its antigenotoxic potential, TBSE demonstrated a statistically significant protective effect against MMC-induced DNA damage at the lowest concentration (0.1 μL/mL), where MN frequency was drastically reduced to 29.5‰ compared to 107‰ of the MMC-treated cells. However, this protective effect was not present at the two highest concentrations (Table 4).

In terms of cytotoxicity, TBSH significantly reduced the CBPI value only at the highest tested concentration (0.5 μL/mL) compared to the control culture. Regarding the mixtures of TBSH with MMC, a slight decrease was noted for the two lowest concentrations, while the highest concentration led to a severe reduction in cell division (CBPI 1.31), which was significantly lower than MMC alone. A slightly higher MN induction was observed for all concentrations tested, which was not statistically significant in any case. TBSH exhibited a distinct biological profile compared to TBSE in terms of antigenotoxicity. All TBSH-MMC mixtures failed to diminish MMC-mediated genotoxic damage. Notably, co-treatment with MMC resulted in an increase in MN frequency compared to the positive control which was statistically significant at 0.1 and 0.5 μL/mL, indicating a synergistic genotoxic effect.

#### 2.3.2. Correlation Analysis Between Cytotoxicity and Genotoxicity of Tonka Bean Extracts

**Figure 2:**
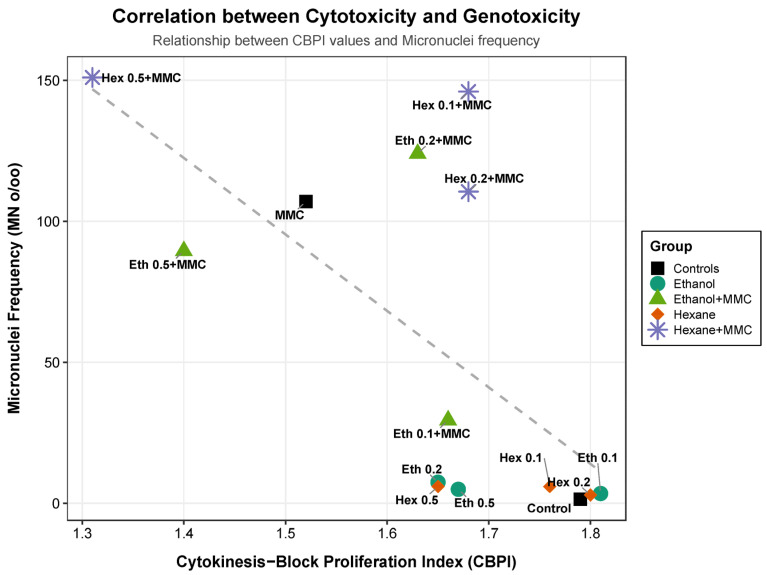
Correlation analysis between Cytokinesis-Block Proliferation Index (CBPI) and Micronuclei (MN) frequency in human lymphocytes treated with tonka bean extracts. Description: The scatter plot illustrates the negative correlation between cell proliferation (x-axis) and genotoxic damage (y-axis). The dashed line represents a linear regression trend. Different shapes and colors indicate the experimental groups: Black squares/circles: Controls (negative and MMC); Green shades: ethanol extract alone or with MMC; Orange/Purple shades: hexane extract alone or with MMC.

To further investigate the relationship between cell proliferation and chromosomal damage, a correlation analysis was performed by plotting the CBPI values against the MN frequency for all tested conditions (Figure 2). As illustrated in the scatter plot, a strong negative correlation was observed (R^2^ = 0.71), indicating that increased genotoxic damage is generally associated with a reduction in cell proliferation.

The distribution of the data points revealed distinct biological profiles. The positive control (MMC) exhibited high genotoxicity and moderate cytotoxicity. The hexane extract in combination with MMC (particularly at 0.5 μL/mL) clustered in the upper-left quadrant, exhibiting the highest MN frequency and lowest CBPI, indicative of a synergistic toxic effect. Conversely, co-treatment with the ethanol extract (0.1 μL/mL) and MMC shifted the experimental point towards the lower right quadrant (closer to the negative control), visually confirming its capacity to simultaneously reduce DNA damage and restore cell proliferation.

## 3. Discussion

### 3.1. GC-MS Analysis of Tonka Bean Extracts

TBSE and TBSH compounds were identified using GC-MS analysis, and the observed variations were mainly attributed to the different polarities of the two solvents. Ethanol, as a polar protic solvent, is highly effective in extracting polar and semi-polar secondary metabolites [20]. Tonka beans are rich in such compounds, with coumarin as the predominant constituent, which was also the case in the present study for TBSE (90.31% area) (Table 1). Similarly, Sousa et al. (2022) [21] identified coumarin as the main compound (87.3% area) in *D. odorata* seed ethanolic extracts using thin-layer chromatography (TLC). Oleic acid was the second most prominent compound in TBSE with a relative area of 4.273%. This is consistent with the work of Fetzer et al. (2022) [22] and Cuchet et al. (2024) [7] where oleic acid was the major fatty acid detected. Furthermore, they have been found to contain phenolic compounds, including flavonoids and phenolic acids [12]. Specifically, o-coumaric acid has been identified in tonka bean extracts alongside coumarin and umbelliferone [13].

In the case of TBSH, coumarin was also the major constituent (Table 1), accounting for approximately 83.5% of the relative area, whereas oleic acid was once again the second most abundant compound with an area of 11.89%—larger than that of TBSE—due to hexane which mostly extracts lipophilic substances as a non-polar solvent [23].

It is noteworthy to mention that the chemical composition of plant extracts is influenced by several factors, including the collection season, climate factors, and the solvent mixture and method used during the extraction process [24,25].

### 3.2. Antimicrobial Activity of Tonka Bean Extracts

A substantial difference was observed in the antimicrobial activity between ethanol (TBSE) and hexane (TBSH) extracts. TBSE possesses strong and broad-spectrum antibacterial activity, with remarkably low MIC values of 15.6 mg/mL against Gram-negative (*E. coli*, *P. aeruginosa*) and Gram-positive (*B. licheniformis*) bacteria, and 62.5 mg/mL against *S. epidermidis*. In contrast, TBSH was active only against *E. coli* and showed weak activity against *P. aeruginosa*. This disparity provides experimental evidence for the nature of the active compounds. Ethanol, a polar protic solvent, is extremely effective in extracting polar and semi-polar secondary metabolites [20]. Tonka beans are rich in such compounds, with coumarin being dominant, as well as in a complex of other phenolic compounds, such as flavonoids and phenolic acids. Hexane, a purely non-polar solvent, preferentially extracts lipophilic substances such as lipids, and other non-polar components [26]. Thus, it is strongly suggested that the lipidic components of the tonka beans lack significant antimicrobial properties, at least against the strains examined.

This observation is particularly important, because Gram-negative bacteria pose a challenge in antimicrobial research [27]. Their outer membrane, rich in lipopolysaccharides, functions as an effective permeability barrier, hindering the entry of many antibiotics and phytochemical compounds into the cytoplasm [28]. Thus, the extract’s ability to overcome this defensive mechanism and exert such potent inhibitory action suggests the presence of compounds with specialized and highly effective mechanisms of action that warrant further investigation.

Furthermore, the selection of the Soxhlet extraction method likely contributed to the high activity observed, as it ensures the maximum possible recovery of soluble compounds from the plant material [29], leading to higher yields and extracts with a higher concentration of active substances compared to other extraction techniques [30].

Our findings contrast with a study by Sousa et al. (2022) [21], which reported weak activity (MIC > 2000 µg/mL) for *D. odorata* seed extracts against *E. coli* and *P. aeruginosa*. Various factors can contribute to this divergence including geographical origin and genotypic diversity, efficiency of the extraction method and differences in the susceptibility of bacterial strains.

A notable methodological observation was the discrepancy between broth micro-dilution and disk diffusion assays. This contradiction constitutes a classic and well-documented phenomenon when testing natural products using agar diffusion based methods [31,32]. Indeed, plant compounds with high molecular weights and/or hydro-phobic or lipophilic properties diffuse poorly through agar, leading to false-negative results in diffusion-based methods [33,34,35].

The GC-MS analysis of our extracts revealed a high content of coumarin and fatty acids (e.g., oleic acid), which are known for their lipophilic nature and poor solubility in the aqueous environment of the agar matrix [22]. Consequently, between the two methods tested in this study, broth microdilution (MIC) is the appropriate method for evaluating the antimicrobial potential of such oily extracts, as it bypasses the diffusion limitations that lead to false-negative results in the disc diffusion assay [22,31,35].

The broad-spectrum efficacy of TBSE is unlikely to be due to a single compound but rather suggests phytochemical synergy, a fundamental advantage of complex extracts [36]. Based on the known phytochemical profile of tonka beans, a plausible synergistic model has been proposed. Flavonoids, known for their membrane-disruptive activity [37,38], can act as permeabilizing agents, destabilizing the outer membrane of Gram-negative bacteria and facilitating the entry of other compounds. Coumarins, the dominant constituents, have been proven to inhibit bacterial DNA gyrase [39], an essential enzyme for the replication, repair, and maintenance of bacterial DNA structure. Aminocoumarins specifically bind to the B subunit of the enzyme (GyrB), inhibiting its ATPase activity and impeding its function [39,40,41]. Studies on *P. aeruginosa* have shown that GyrB inhibitors are effective [42]. Moreover, coumarins have been reported to disrupt cellular membrane integrity [43], inhibiting the quorum sensing system in *P. aeruginosa*, a communication mechanism regulating virulence and biofilm formation [44].

In conclusion, TBSE exhibits promising, broad-spectrum antibacterial activity driven by polar phenolic compounds. This study also methodologically emphasizes the importance of standardizing plant material origin and methodological protocols to achieve reproducible and comparable results.

### 3.3. CBMN Assay in Human Lymphocytes

The cyto-genotoxic and antigenotoxic evaluation of tonka bean extracts addresses a critical gap in the literature. Despite their widespread use in food and cosmetics, no previous study has assessed either the genotoxic risk of whole tonka bean extracts, or their antigenotoxic potential. This is particularly significant because the effects of complex natural mixtures often differ from those of their isolated constituents due to synergistic or antagonistic interactions among components.

#### 3.3.1. Cytotoxic Activity of Tonka Bean Extracts

Lack of cytotoxicity was reported for the lowest dose of TBSE, while 0.2 and 0.5 μL/mL reduced CBPI, albeit in a mild manner. In the case of TBSH, statistically significant cytotoxic induction was observed only at the highest concentration. Notably, tonka beans and/or their extracts have not been investigated regarding their cytotoxic profile. Research focuses largely on natural and synthetic coumarins and their biological activities [45,46]. To this end, studies regarding coumarin—the main constituent of tonka beans—can constitute a basis to explain our findings to an extent.

Coumarins possess several characteristics, including solubility in organic solvents, simple molecular structures, low molecular weight and high bioavailability, which coupled with their low toxicity, contribute to their diverse biological activities [47]. The cytotoxic induction observed at higher concentrations of TBSE and TBSH could partly be due to the presence of coumarin, as in vitro and in vivo studies have confirmed the cytotoxic effects of coumarins on different cancer cell lines [48,49] in addition to their cytostatic properties [50]. Some of their most prominent modes of action include induction of cell apoptosis, hindrance of tumor multidrug resistance, and inhibition of angiogenesis [45]. According to Chuang et al. (2007) [51], coumarin’s efficacy against HeLa cells was due to inducing cell cycle arrest at the G0/G1 phase and apoptosis, through mitochondria and caspase-3 dependent mechanisms and via the downregulation of NF-κB proteins [51]. Moreover, the anticancer activity of coumarin in Hep2 cells has been attributed to apoptosis induction and DNA fragmentation [52]. Umbelliferone—another naturally occurring coumarin—inhibits the release of cyclin D1, hindering its overexpression which can lead to cancer development [53]. Similarly, it induced apoptosis through cell cycle arrest at the S phase and DNA fragmentation when its effect was tested on HepG2 cells [54]. The mild cytotoxicity observed in the higher doses and lack of it in the lowest in the present study, could be due to the use of non-cancerous cells, i.e., human lymphocytes, which could be exploited for future medical treatments and applications. Moreover, even though coumarin is the main ingredient of tonka bean extracts, they possess several other compounds—polyphenols, flavones, lipid acids—which could lead to a different outcome.

In the presence of MMC, a statistically significant reduction compared to the negative control was observed in all cases for both mixtures, which was mild in the case of the two lowest concentrations, i.e., 0.1 and 0.2 μL/mL, respectively, whereas the highest had a CBPI of 1.4 and 1.31 in the case of TBSE and TBSH, respectively, with approximately 50 and 60% cytostasis. Notably, the CBPI of the two lowest concentrations was significantly higher than that of MMC alone, showcasing the potential protective effect of the extracts against MMC-mediated cytotoxicity in human lymphocytes. The cytoprotective role of natural products and/or their extracts has been noted in numerous studies and is usually attributed to their phytochemical profile and antioxidant properties. Our findings can be corroborated by Al-Naqeb et al. (2024) [55], who determined among others the potential cytoprotective effects of *Cistus monspeliensis* L. leaf extract against MMC using the in vitro CBMN assay in the Chinese Hamster Ovarian K1 (CHO-K1) cell line. Cells treated with the highest concentration of the extract (200 μg/mL) combined with MMC demonstrated a significant increase in cytotoxicity compared to those treated with MMC alone. In contrast, the lowest concentration of the extract (50 μg/mL) coupled with MMC induced a protective effect against MMC cytotoxicity. Phenolic compounds from *Codiaeum variegatum spirale* leaves were tested against the cytotoxic effects of MMC in bone marrow cells of male Swiss albino mice. Interestingly, the percentage of viable cells in mice treated with MMC reached approximately 70%, whereas the percentage ranged from 75 to 86% in mice simultaneously treated with MMC and the plant’s phenolic compounds [56].

#### 3.3.2. Genotoxic and Antigenotoxic Activity of Tonka Bean Extracts

In terms of genotoxicity, TBSE induced a statistically significant increase in micronuclei (MN) frequency only at 0.2 μL/mL, with slightly higher MN values compared to the negative control. A lack of genotoxic activity was reported for the remaining concentrations. In the case of TBSH, a slightly higher MN induction was observed for all concentrations tested, which was not statistically significant in any case. Similar to cytotoxicity, the genotoxic induction of tonka beans has not been investigated. Thus, research on its major component, coumarin, was considered.

Most studies have reported a lack of genotoxicity of coumarins. According to Api AM (2001) [57] coumarin did not induce genotoxic damage in male and female Swiss mice when the in vivo micronucleus assay was applied. Similar results were reported when coumarin’s genotoxic profile was assessed using the unscheduled DNA synthesis (UDS) assay in male Sprague Dawley CD rat hepatocytes in vivo [58]. In contrast, coumarin exerted genotoxic and biochemical damage on *Lens culinaris* Medik, as demonstrated by the alteration of key biochemical parameters and gene expression [59].

Although, TBSE and TBSH followed a somewhat similar pattern regarding their cyto-genotoxic activity, a distinctly different biological profile was observed for TBSE compared to TBSH regarding their antigenotoxic potential. Regarding its antigenotoxic potential, TBSE demonstrated a striking protective effect against MMC-induced DNA damage at the lowest concentration (0.1 μL/mL). Coumarins possess significant antioxidant properties that operate through free radical scavenging [60], stimulating the activity of antioxidant enzymes [61], and modulating transcription factors involved in oxidative stress [62]. Fetzer et al. (2022) [22] performed ethanolic extraction of *D. Odorata* seeds using the Soxhlet method and investigated their antioxidant activity and total phenolic content (TPC). The findings revealed that the ethanolic extract exhibited significant antioxidant activity with DPPH• and ABTS values of 902 and 443 nmol TE/100 g, respectively, and a TPC value of approximately 112 mg GAE/100 g. However, the protective effect of TBSE was not present at the two highest concentrations. The antigenotoxicity demonstrated only by the lowest concentration of TBSE, can be explained through hormesis, a phenomenon describing the ability of a compound to induce opposite effects at different doses, which is widely observed in phytochemicals [63]. Similar results have been reported previously using the same assay [64,65].

All mixtures of TBSH with MMC failed to diminish the MMC-mediated genotoxic damage. Notably, co-treatment with MMC resulted in increased MN frequency compared to the positive control, indicating a synergistic genotoxic effect. Coumarins have been found to act as both antioxidants and pro-oxidants, usually exerting antioxidant activity at lower concentrations while leading to increased ROS production at higher doses [66].

The different profiles of TBSE and TBSH can be attributed to the different solvents used for extraction. Ethanol, a polar solvent, can efficiently extract various compounds, especially bioactive substances including phenolics, flavonoids, and fatty acids [18]. Hexane is a non-polar solvent that leads to the extraction of lipophilic substances such as lipids, terpenoids, and essential oils [67]. This distinct difference in the composition of the extracts leads to prominent variations in their bioactivities. Fetzer et al. (2018) [68] performed baru (*Dipteryx alata* vogel) seed oil extraction via Soxhlet method using hexane and ethanol as solvents. The hexane extract had the lowest antioxidant activity as demonstrated by the ABTS assay, with a value of 9.98 μM de trolox/g, whereas the ethanolic extract had a value of 23.20 μM de trolox/g.

According to our results it can be inferred that the lowest concentration of TBSE (0.1 μL/mL) should be further investigated to be used in various applications, since it seems to abide to safety standards and possesses significant antigenotoxic potential.

#### 3.3.3. Correlation Analysis Between Cytotoxicity and Genotoxicity of Tonka Bean Extracts

A clear correlation was depicted between increased MN frequency and reduced CBPI, especially at the highest concentration of TBSH-MMC mixture, indicating simultaneous cell viability decrease and significant DNA damage. Even though, cell death could contribute to the significant genotoxic activity, the different pattern exhibited at the same concentration of TBSE-MMC mixture indicates additional reasons and mechanisms at play. In fact, even though cytotoxicity was also quite high in this case, MN induction is remarkably lower than that observed at the TBSH-MMC mixture.

In contrast, relatively high cell proliferation is noted paired with significantly lower MN induction in the case of the lowest concentration of TBSE-MMC mixture (0.1 μL/mL). The higher antioxidant activity of TBHE, as previously mentioned [22], could correlate with the low genotoxic potential due to free radical scavenging combined with possible DNA repair mechanisms.

## 4. Materials and Methods

### 4.1. Reagents

Ham’s F-10 medium, Foetal Bovine Serum (FBS), and Phytohaemaglutinin (PHA) were commercially procured from Gibco (Scotland, UK). Mitomycin C (MMC) and Giemsa stain were purchased from Sigma-Aldrich Chemical Co. (St. Louis, MO, USA). Cytochalasin-B (Cyt-B) was obtained from Santa Cruz Biotechnology (Dallas, TX, USA), while Resazurin was acquired from Cayman Chemical Company (Ann Arbor, MI, USA) and from Fisher Chemical (Loughborough, UK). Regarding the microbiological assays, Mueller-Hinton Agar (MHA) and Mueller-Hinton Broth (MHB) were provided by Thermo Scientific™ (Waltham, MA, USA). Finally, sterile paper discs (6 mm diameter) were obtained from Mast Group (Merseyside, UK, Cat. No. BD0680W/C/NCE), 96-well microtiter plates were purchased from Thermo Scientific, Cat. No. 167008 (Roskilde, Denmark) and DMSO from Fisher Chemical, Loughborough, UK (Cat. No. D/4121/PB15).

### 4.2. Sample Preparation

Tonka beans (*Dipteryx odorata* [Aubl.] Willd) were collected in Altamira City, state of Pará, Brazil, and the average particle size distribution was reduced to 2.0 ± 0.1 mm. The moisture content and volatile compounds were determined according to AOAC methods 925.40 (2005) [69]. The sample was stored in a −5 °C freezer inside a vacuum-packed polypropylene plastic bag until use.

### 4.3. Soxhlet Extraction (SE)

Soxhlet extraction (SE) of tonka beans was performed using n-Hexane (99.8% purity, Neon, Brazil) and ethanol (99.8%, Neon, Brazil) as solvents. Approximately 5 g of tonka beans were placed in a cellulose cell and loaded into a Soxhlet apparatus (UNIGLAS, Campinas, São Paulo, Brazil). SE were conducted with 150 mL of solvent under reflux conditions, where the temperature was close to the boiling point of the solvent, according to the 920.39C method of AOAC [70], with some modifications. Afterward, the solvent was removed from the oil using a rotary vacuum evaporator (IKA, Model RV 10 digital, Staufen im Breisgau, Germany). SE showed a similar yield to that obtained by Fetzer et al. (2022; 2018) [22,68].

### 4.4. GC-MS Analysis

Chromatography analysis was carried out in a GC-MS (QP-2010 model, Shimadzu Sc entific Instruments, Kyoto, Japan) equipped with a split/splitless auto-injector model AOC-20i, an auto sampler model AOC-20s and a capillary column (HT8, 25 m, 0.22 mm diameter and 0.25 μm thickness, SGE by Trajan Scientific and Medical, Ringwood, Victoria, Australia). The carrier gas was helium at a flow rate of 1.43 mL/min and injector temperature of 280 °C. The oven temperature program was 50 °C (held for 1 min) and then raised to 280 °C with 4 °C /min, where the program was completed. The temperatures of the ionic source, and interface were set at 230 °C and 280 °C, respectively. Chromatograms were acquired in scan mode from 50 to 800 m/z. The compounds were identified via mass spectra comparison with the NIST27 and NIST147 mass spectral libraries (minimum similarity of 75%).

### 4.5. Antimicrobial Activity

#### 4.5.1. Bacterial Strains

To assess the antimicrobial activity of TBSE and TBSH, Gram-positive *Staphylococcus epidermidis* (DSM 20044), *Bacillus licheniformis* (DSM 10) and Gram-negative *Pseudomonas aeruginosa* (DSM 50071), *Escherichia coli* (DSM 30083) were used. All strains were acquired from the German Collection of Microorganisms and Cell Cultures (Leibniz Institute, Berlin, Germany).

#### 4.5.2. Culture Media and Reagents

For the culture of the strains, Mueller-Hinton Agar (MHA) (Thermo Scientific™, Massachusetts, Waltham, MA, USA, Cat. No. OXCM337B) and Mueller-Hinton Broth (MHB) (Thermo Scientific™, Massachusetts, USA, Cat. No. OXCM0405B) were used for solid and liquid cultures consecutively.

A 0.015% (*w*/*v*) solution of resazurin (Cheyman Chemical Company, Ann Arbor, MI, USA, Cat. No. 14322) was prepared in sterile distilled water. The solution was filtered through a sterile 0.22 μm filter and stored at 4 °C for up to two weeks.

#### 4.5.3. Antimicrobial Activity Evaluation by the Kirby-Bauer Disc Diffusion Method

The agar disc diffusion method was used for an initial, qualitative evaluation of the antimicrobial activity of the extract. After 18–24 h of cultivation of the bacterial strains on non-selective agar, a suspension was prepared in sterile saline. The turbidity of the suspension was adjusted to correspond to the 0.5 McFarland standard (approximately 1−2 × 10^8^ CFU/mL). Within 15 min of its preparation, the adjusted suspension was uniformly coated on the surface of MHA plates using a sterile swab, to achieve the creation of a uniform bacterial lawn. Sterile paper discs (6 mm diameter, Mast Group, Merseyside, UK) were impregnated with the pure extract (100% *v*/*v* oil) to saturation (approximately 20 µL per disc) and placed on the inoculated agar. The discs were placed at least 24 mm apart from each other. The plates were inverted and incubated at 35 ± 2 °C for 16–18 h. After incubation, the diameter of the zone of inhibition (clear zone around the plate where no growth was observed) was measured in millimeters (mm).

#### 4.5.4. Determination of Minimum Inhibitory Concentration (MIC) by Resazurin Microdilution and Staining Method

The Minimum Inhibitory Concentration (MIC), i.e., the lowest concentration of the extract that visibly inhibits the growth of the microorganism, was determined by the microdilution method in 96-well microplates, using resazurin as a viability indicator. The protocol used was based on the protocol of Mohamed Elshikh et al. (2016) [71].

The 96-well microplate was prepared as follows: 100 μL of sterile MHB medium was added to column 12 as a negative control. For the positive control (column 11), 50 μL of sterile MHB was combined with 50 μL of the adjusted bacterial inoculum. This ensured that the final volume (100 μL) and bacterial density were consistent with the test wells (which contained 50 μL extract + 50 μL inoculum). 100 μL of the original extract solution was added to column 1 and 50 μL of sterile MHB was added to columns 2 to 10. Serial 1:2 dilutions of the extract were performed, transferring 50 μL from column 1 to column 2, mixing, and repeating the process up to column 10, from which the last 50 μL were discarded. 50 μL of the adjusted bacterial inoculum (OD_600_ ≈ 0.14) was added to the prepared wells, so that the final volume in each well was 100 μL. An incubation of 18 h was followed at 37 °C. After the incubation period, 50 μL of resazurin solution (0.015%) was added to all wells (columns 1–12) and the plate was incubated for an additional 2–3 h at 37 °C. The MIC value was determined as the lowest concentration of the extract that prevented the indicator color from changing from purple (absence of growth) to pink (presence of growth). To ensure reproducibility, MIC values were converted from volume percentages (% *v*/*v*) to mass concentrations (mg/mL) assuming a standard extract density of 1.0 g/mL.

### 4.6. CBMN Assay

#### 4.6.1. Ethics Statement

Peripheral blood lymphocytes were obtained from two healthy, non-smoking male volunteers under 30 years of age, who were not exposed to radiation, pharmacological treatment, or viral infection in the recent past. Prior to sample collection, written informed consent was secured, and the study was conducted with the approval of the Research Ethics Committee of the University of Patras (Ref. No 7530/24-02-2022), following international bioethical criteria.

#### 4.6.2. CBMN Assay Application

The assay was performed according to OECD 487 [72]. Cultures were initiated with 0.5 mL whole blood in 6.5 mL Ham’s F-10 medium, supplemented with 1.5 mL FBS and 0.3 mL PHA. Treatments with ethanol and hexane tonka bean extracts (0.1, 0.2, 0.5 µL/mL), with or without MMC (0.5 µg/mL), were applied 24 h later. Cytochalasin-B (6 µg/mL final concentration) was introduced at 44 h post-initiation [73]. Cultures were grown for 72 h at 37 °C under 5% CO_2_.

Following incubation, cells were harvested by centrifugation (1500 rpm, 10 min). They were subjected to a mild hypotonic treatment (Ham’s medium: Milli-Q water, 3:1 ratio) at room temperature and fixed in triplicate with freshly prepared methanol/acetic acid (5:1 ratio). Finally, slides were prepared by staining with 10% (*v*/*v*) Giemsa. The entire procedure was performed in duplicate for each condition.

The CBPI was derived from a minimum of 1000 cells per data point using the published formula: CBPI = [N1 + 2N2 + 3(N3 + N4)]/N, where N1, N2, N3 and N4 correspond to the numbers of cells with one, two, three, and four nuclei and N is the total number of cells [74]. MN frequency was evaluated automatically by the Metafer MNScore platform, scanning no fewer than 2000 BN cells per concentration against established cytogenetic criteria [75,76].

### 4.7. Statistical Analysis

Data are expressed as Mean ± Standard Error (S.E.) of at least two independent experiments. Statistical analysis was performed using IBM SPSS Statistics for Windows (Version 25.0; IBM Corp., Armonk, NY, USA). The normality of the data distribution was verified using the Shapiro–Wilk test. Statistical significance was assessed using One-Way Analysis of Variance (ANOVA), followed by Dunnett’s multiple comparison test. Comparisons were conducted against the negative control to evaluate genotoxicity and against the positive control (MMC) to evaluate antigenotoxicity. Differences were considered statistically significant at *p* < 0.05.

Additionally, a correlation analysis was performed to examine the relationship between cytotoxicity (CBPI) and genotoxicity (MN frequency). The scatter plot and linear regression analysis were generated using the ‘ggplot2’ package (version 3.5.1) in R statistical software (version 4.4.0) (R Foundation for Statistical Computing, Vienna, Austria).

## 5. Conclusions

*D. odorata* seeds, known mainly as tonka beans, are widely used in gastronomy, cosmetics and the perfume industry due to their characteristic odor. Despite their numerous applications, research on tonka beans remains scarce, with most studies focusing on the phytochemical characterization and bioactivity of their main compound, coumarin. The present study attempted to fill this significant gap through the holistic investigation of ethanol and hexane extracts. The ethanolic extract exhibited significant antimicrobial activity against both Gram-positive and Gram-negative bacteria. That was not, however, the case for the hexane extract which was active only against *E. coli* and showed weak or no activity against the other three strains. To our knowledge, this is the first study to evaluate the cytotoxic, genotoxic and antigenotoxic profiles of tonka bean extracts. Both extracts were cytotoxic at the highest concentrations but mostly lacked genotoxic activity. The ethanol extract at the lowest dose exhibited remarkable antigenotoxic potential. The hexane extract lacked antigenotoxic potential and increased MMC mediated genotoxicity. These findings could prove crucial for further applications in pharmaceutics and medicine, as this study could constitute the starting point for further research on the antimicrobial and antigenotoxic potential of tonka beans. Future studies should investigate the specific compounds responsible for the observed antigenotoxic activity, explore dose–response relationships across a wider concentration range, and evaluate the extracts’ efficacy in relevant in vivo models. Additionally, the development of extraction protocols that maximize beneficial compound recovery while minimizing coumarin content could enhance the safety profile for potential therapeutic applications.

## Figures and Tables

**Table 1:** Compounds identified in the ethanol (TBSE) and hexane (TBSH) tonka bean extracts using GC-MS.

No	RT (min)	MW (g/mol)	Molecular Formula	% Area	Compound
TBSE					
1	29.44	146.14	C_9_H_6_O_2_	90.31	Coumarin
2	40.43	709.05	C_42_H_76_O_8_	0.29	L-Ascorbic acid 2,6-dihexadecanoate
3	22.00	186.33	C_12_H_26_O	0.09	2-Butyl-1-octanol
4	23.93	252.5	C_18_H_36_	0.02	5-Octadecene,
5	26.21	298.55	C_20_H_42_O	0.16	Decane, 1,1′-oxybis-
6	24.16	252.5	C_18_H_36_	0.2	9-Octadecene, (E)-
7	24.384	226.44	C_16_H_34_	0.095	Hexadecane
8	32.475	142.24	C_9_H_18_O	0.032	Nonanal
9	45.32	282.5	C_18_H_34_O_2_	4.273	Oleic Acid
TBSH					
1	29.36	146.14	C_9_H_6_O_2_	83.969	Coumarin
2	40.43	709.05	C_42_H_76_O_8_	1.284	L-Ascorbic acid 2,6-dihexadecanoate
3	22.01	186.33	C_12_H_26_O	0.128	2-Butyl-1-octanol
4	23.92	280.50	C_20_H_40_	0.051	5-Eicosene, (E)-
5	26.22	298.55	C_20_H_42_O	0.295	Decane, 1,1′-oxybis-
6	24.16	252.5	C_18_H_36_	0.410	9-Octadecene, (E)-
7	24.375	226.44	C_16_H_34_	0.189	Hexadecane
8	44.089	280.40	C_18_H_32_O_2_	0.032	Linoleic acid
9	45.573	282.5	C_18_H_34_O_2_	11.89	Oleic Acid

**Table 2:** Inhibition zone diameters (mm) of tonka bean extracts against bacterial strains. Values are expressed as the mean ± standard deviation.

Extract	*E. coli*	*P. aeruginosa*	*B. licheniformis*	*S. epidermidis*
TBSE (Ethanol)	18.0 ± 0.0	11.5 ± 0.71	0.0 ± 0.0	0.0 ± 0.0
TBSH (Hexane)	0.0 ± 0.0	0.0 ± 0.0	0.0 ± 0.0	0.0 ± 0.0

**Table 3:** Antimicrobial activity and Minimum Inhibitory Concentration (MIC) of ethanol and hexane oil extracts. Data represents the Mean ± Standard Error of the Mean (SEM) of independent replicates. Disc diffusion results are reported as the diameter of the inhibition zone (mm). MIC values are presented as mass concentration (mg/mL), derived from volume percentage for an oil extract of 1.0 mg/mL.

Bacterial Strain	Extract	Average Inhibition Zone Diameter (mm)	MIC Value (mg/mL)
*E. coli*	TBSE	18.0 ± 0.0	15.6 ± 0.0
	TBSH	0.0 ± 0.0	125.0 ± 0.0
*P. aeruginosa*	TBSE	11.5 ± 0.7	15.6 ± 0.0
	TBSH	0.0 ± 0.0	500 ± 0.0
*B. licheniformis*	TBSE	0.0 ± 0.0	15.6 ± 0.0
	TBSH	0.0 ± 0.0	>500 ± 0.0
*S. epidermidis*	TBSE	0.0 ± 0.0	62.5 ± 0.0
	TBSH	0.0 ± 0.0	>500 ± 0.0

**Table 4:** Frequency of micronuclei (MN), Cytokinesis-Block Proliferation Index (CBPI) and % Cytostasis in cultured human lymphocytes treated with tonka bean extracts and/or Mitomycin-C (MMC).

Treatment	MN (MF ‰ ± se)	CPBI (MF ‰ ± se)	Cytostasis (%)
Control	1.5 ± 0.8	1.79 ± 0.02 ^#^	0
MMC (0.5 μg/mL)	107.0 ± 5.7 *	1.52 ± 0.02 *	34.1 ± 2.5
	TBSE (μL/mL)		
0.1	3.5 ± 0.71	1.81 ± 0.00 ^#^	-
0.2	7.5 ± 1.41 *	1.65 ± 0.04 *^#^	17.5 ± 3.6
0.5	5.0 ± 0.0	1.67 ± 0.01 *^#^	15.6 ± 1.0
	TBSE + MMC		
0.1 + MMC	29.5 ± 6.4 *^#^	1.66 ± 0.08 *^#^	16.7 ± 9.1
0.2 + MMC	124.0 ± 4.2 *^#^	1.63 ± 0.08 *^#^	20.6 ± 5.4
0.5 + MMC	89.5 ± 7.8 *	1.40 ± 0.01 *^#^	50.6 ± 1.3
	TBSH (μL/mL)		
0.1	6.0 ± 0.7 ^#^	1.76 ± 0.03 ^#^	2.5 ± 1.0
0.2	3.0 ± 0.0 ^#^	1.80 ± 0.04 ^#^	-
0.5	6.0 ± 1.4 ^#^	1.65 ± 0.01 *^#^	18.3 ± 1.0
	TBSH + MMC		
0.1 + MMC	146.0 ± 7.0 *^#^	1.68 ± 0.01 *^#^	14.5 ± 2.0
0.2 + MMC	110.5 ± 3.5 *	1.68 ± 0.06 *^#^	15.0 ± 1.0
0.5 + MMC	151.0 ± 5.0 *^#^	1.31 ± 0.03 *^#^	60.1 ± 1.4

MN: micronuclei; se: standard error; MMC: mitomycin C (positive control); CBPI: Cytokinesis Block Prolifiration Index; MF (‰) ± se: mean frequencies (‰) ± standard error; Statistical analysis was performed using One-Way ANOVA followed by Dunnett’s multiple comparison test. MN were scored in 2000 binucleated lymphocytes per experimental point. * Indicates statistically significant difference compared to the negative control (*p* < 0.05). ^#^ Indicates statistically significant difference compared to the positive control (MMC 0.5 μg/mL) (*p* < 0.05).

## Data Availability

The original contributions presented in this study are included in the article. Further inquiries can be directed to the corresponding author.

## Abbreviations

The following abbreviations are used in this manuscript:

TBSE	Tonka bean Soxhlet Ethanol extract
TBSH	Tonka bean Soxhlet hexane extract
GC-MS	Gas Chromatography—Mass Spectrometry
DDT	Disc Diffusion Test
MIC	Minimum Inhibitory Concentrations
CBMN	Cytokinesis-Block Micronucleus
MMC	Mitomycin C
CBPI	Cytokinesis Block Proliferation Index
MN	Micronuclei
